# Altered expression of inflammation-associated molecules in striatum: an implication for sensitivity to heavy ion radiations

**DOI:** 10.3389/fncel.2023.1252958

**Published:** 2023-12-01

**Authors:** Zixuan Chen, Yumeng Li, Madiha Rasheed, Hao Wang, Runhong Lei, Tuo Zhao, Yulin Deng, Hong Ma

**Affiliations:** ^1^Beijing Key Laboratory for Separation and Analysis in Biomedicine and Pharmaceuticals, School of Medical Technology, Beijing Institute of Technology, Beijing, China; ^2^Department of Radiation Oncology, Peking University Third Hospital, Beijing, China

**Keywords:** heavy ion radiations, striatum, immune system, neuroinflammation, astrocytes

## Abstract

**Background and objective:**

Heavy ion radiation is one of the major hazards astronauts face during space expeditions, adversely affecting the central nervous system. Radiation causes severe damage to sensitive brain regions, especially the striatum, resulting in cognitive impairment and other physiological issues in astronauts. However, the intensity of brain damage and associated underlying molecular pathological mechanisms mediated by heavy ion radiation are still unknown. The present study is aimed to identify the damaging effect of heavy ion radiation on the striatum and associated underlying pathological mechanisms.

**Materials and methods:**

Two parallel cohorts of rats were exposed to radiation in multiple doses and times. Cohort I was exposed to 15 Gy of ^12^C^6+^ ions radiation, whereas cohort II was exposed to 3.4 Gy and 8 Gy with ^56^Fe^26+^ ions irradiation. Physiological and behavioural tests were performed, followed by ^18^F-FDG-PET scans, transcriptomics analysis of the striatum, and *in-vitro* studies to verify the interconnection between immune cells and neurons.

**Results:**

Both cohorts revealed more persistent striatum dysfunction than other brain regions under heavy ion radiation at multiple doses and time, exposed by physiological, behavioural, and ^18^F-FDG-PET scans. Transcriptomic analysis revealed that striatum dysfunction is linked with an abnormal immune system. *In vitro* studies demonstrated that radiation mediated diversified effects on different immune cells and sustained monocyte viability but inhibited its differentiation and migration, leading to chronic neuroinflammation in the striatum and might affect other associated brain regions.

**Conclusion:**

Our findings suggest that striatum dysfunction under heavy ion radiation activates abnormal immune systems, leading to chronic neuroinflammation and neuronal injury.

## Introduction

With the rapid development of space life science, the number of astronauts for space exploration has increased, hence necessitating the assessment of the risk of exposure to cosmic radiation. Space radiation is a major harmful environmental factor that may endanger astronaut’s health during long-term flights (Zeitlin et al., 2013). Heavy ion irradiation, a crucial high-linear energy transfer space particle, has a higher biological effect and results in short-term and long-term physiological and pathological changes (Furukawa et al., 2020). Extensive evidence shows that heavy radiation induces brain injury, including irreversible cerebral spinal cord disorders, white matter necrosis, and impaired hippocampal neurogenesis and memory (Kishimoto et al., 2005; Manda et al., 2008; Cacao and Cucinotta, 2016). The central nervous system (CNS) damage induced by whole-brain irradiation can be divided into acute, early, and late-delayed stages. The clinical pathological effects of CNS induced by components of space-heavy ion irradiation show a diversity of neural malfunctions at different exposure stages, manifesting by brain oedema, neuroinflammation, chromatin dissolved at the acute stage, vacuoles degeneration, swelling of nerve fibres, and demyelination at an early stage and the brain microvascular circulation disorder, white matter necrosis, and cognitive dysfunction at late-delayed stages (Du et al., 2014; Cucinotta, 2015). Despite an intensive study of these neural abnormalities, the pathomechanism of sensitive brain regions has not been well understood.

The brain is a complex organ with multiple regions, physically or functionally connected networks, mainly including the hypothalamus, striatum, substantia nigra, cortex, and amygdala (Jurkowski et al., 2020). The tremendous biodiversity of cell types with different functions and morphology in different brain regions has been identified, resulting in the CNS’s complex structural and functional composition (Sharma et al., 2015). There is growing evidence that some neurons are more sensitive to environmental stress, probably because of different gene expression patterns in the specific brain subdivisions (Ko et al., 2013; Pfisterer and Khodosevich, 2017). Rauskolb et al. (2010) showed an essential difference in the effect of BDNF on the dendritic architecture of striatal versus hippocampal neurons. In Tau-bdnf ko mice, there are significant reductions in the volume, dendritic length, and complexity of dendritic spine density of the striatum, while not in the cortex and hippocampus (Zagrebelsky et al., 2018).

Striatum consists of neuronal activity associated with movements, rewards, or a combination of both and presents activities related to the preparation, initiation, and execution of movements (Hollerman et al., 2000). Striatal neurons connect with multiple regions and send signals to the cortex, amygdala, and hippocampus (Volman et al., 2013). Abnormalities in the striatum result in motor disabilities that limit signal transduction to the other areas. Pathological studies on various neurological disorders showed that abnormal striatal functioning is triggered by neuroinflammation (Mancini et al., 2021). Surprisingly, an aberrant immune response, including reduced lymphocytes, peripheral immune organs, and immunosuppression, is one of the adverse effects of cosmic radiation in astronauts (Onorato et al., 2020). Thus, it suggests that cosmic radiation might adversely impact the striatum, disrupting CNS functioning. So far, the underlying pathology of the striatum under heavy ion radiation and its consequences are still unknown. Therefore, this study identifies the sensitivity of different brain regions, especially the striatum, and associated underlying pathological mechanisms under heavy ion radiation exposure *in vitro* and *in vivo*. For this purpose, we used two cohorts of SD rats and exposed them to ^12^C^6+^ and ^56^Fe^26+^ radiations, respectively, for multiple time zones (1, 2, and 3 months) and dose ranges (^^12^C^6+^^, 15 Gy, ^56^Fe^26+^, 3.4 Gy, and 8 Gy). After irradiation, rats were tested for physiological and behavioural tests. And then, PET scans were executed to determine abnormal glucose metabolism in brain regions, which showed the striatum as most affected in both cohorts. Furthermore, the transcriptomic analysis revealed that radiation induces abnormal neuroinflammation in the striatum, further verified by irradiating medium cell culture of glial cells and neurons.

## Materials and methods

### Animals

All experimental procedures were performed following the guidelines for animal care of the National Institute of Health. The ethical committee approved all Beijing Institute of Technology procedures and the Key Laboratory of Heavy ion Radiation Biology and Medicine of the Chinese Academy of Sciences. In the present study, parallel experiments were performed on the two cohorts of rats such that cohort I was irradiated with ^^12^C^6+^^ ions and cohort II rats was irradiated with ^56^Fe^26+^ ion for 1, 2, and 3 months to study the impact of radiation on the neurobiology of the striatum. A total of 84 male Sprague Dawley (SD) rats (4 weeks old, weighing 180 ± 10 g) were housed in standard conditions with temperature 22 (±1)°C, humidity, 40–50%, 12 h light and dark cycle with free access to food and water to acclimatise normal conditions. All experimental rats (cohort I, cohort II, and the control group) were given free access to food and water. The control groups were treated like the irradiated experimental group except for irradiation. Feeding conditions remain persistent before and after irradiation.

### Irradiation

Cohort I was subdivided into three experimental groups (G1, G2, G3: 15 Gy of ^12^C^6+^: 1 month, 2 months, and 3 months, respectively), (*n* = 6) per group, and three control groups (C4, C5, C6: 0Gy: 3 months), (*n* = 6) per group. Cohort II was subdivided into four experimental groups (G7, G8, G9: 3.4 Gy of ^56^Fe^26+^: 1, 2, 3 months, respectively, and G10: 8 Gy of ^56^Fe^26+^ for 2 months), (*n* = 6) per group, and four control groups (C11, C12, C13, C14: 0Gy: 3 months), (*n* = 6) per group. The Heavy Ion Research Facility in Lanzhou (HIRFL), Institute of Modern Physics, Chinese Academy of Sciences received rats for irradiation. The animals from both cohorts were weighed, anaesthetised intraperitoneally with 5% pentobarbital (70 μg/kg), and fixed to an irradiation machine at HIRFL. The whole brain of cohort 1 rats was irradiated with a single dose of ^^12^C^6+^^ ions at a distance of 200 cm with an intensity of 0.5 Gy/min for 30 min to achieve 15 Gy total dosage (165 MeV/u primary energy; LET, 30 keV/μm). In cohort II (groups 7–9), animals were irradiated with ^56^Fe^26+^ ion radiations at a distance of 200 cm with an intensity of 0.7 Gy/min for 4.8 min to achieve 3.4 Gy dosage, and group 10 animals were irradiated with ^56^Fe^26+^ ion radiations at a distance of 200 cm with an intensity of 0.7 Gy/min for 11.4 min to achieve 8 Gy dosage (163 MeV/u primary energy, LET, 500–1,000 keV). Control groups of both cohorts were anaesthetised and fixed for the same period without irradiation. All irradiated rats from both cohorts and controls were sacrificed after 1, 2, or 3 months, respectively.

### Behavior assessment

After irradiation, rats of each group from both cohorts were transferred to standard laboratory conditions and rehabilitated for 15 days. Behavioural tests were performed after 15 days to assess depressive-like behaviours. An open-field test was initially performed to determine anxious behaviours (Silverman et al., 2013). Rats were exposed to open fields on the 36th, 76th, and 106th day after irradiation to observe their curiosity levels when exposed to the new environment in an open area or prefer to remain near protective walls. The overall activity of each rat was observed, including total time and distance travelled in the central arena, no movement time, local movements (<10 cm), and average velocity of the moment.

After 24 h of the open field test, a rotarod test was performed to evaluate motor disabilities in rats developed after irradiation, as described previously (Brooks and Dunnett, 2009; Yoshihara et al., 2009). Rats were subjected to the rotarod paradigm on the 38th day, 78th day, and 108th day for three trials per day for 3 successive days with a 300-s accelerating program from 5 to 40 rpm, and abeyance to fall from the rod was measured for analysis. Again, rats were normalised for 24 h, and a sugar preference test was performed to determine the anhedonia level as described earlier (Serchov et al., 2016). Each cohort was subjected to tests on the 41st day, 81st day, and 111th day. Sucrose preference levels between control and irradiated groups were analysed using the formula: Sucrose preference percentage (%) = sucrose intake (ml)/[sucrose intake (ml) + water intake (ml)] × 100%.

### PET imaging

After behavioural tests, rats were normalised for a few days and subjected to PET (Position emission tomography) scanning on the 45th day, 85th day, and 115th day. Rats were fasted for 8–12 h without free access to water and then anaesthetised with isoflurane. One unit of ^18^F-FDG was injected into anaesthetised rats via tail. After 30 min, rats were administered to the imaging device for brain scanning for 15 min, and changes in glucose metabolism were analysed with SPM software.

### Animal sample collection

Each cohort of rats was weighed, deeply anesthetized with pentobarbital sodium (60 mg/kg of body weight, concentration, 20 mg/mL), and then sacrificed. After perfusion with cold saline solution, the striatum, thymus, and spleen were excised on an ice-cold plate then washed with phosphate buffer solution and stored at −80°C for further experimentation. Additionally, thymus, and spleen were weighed before being preserved at −80°C.

### RNA extraction and cDNA library preparation

RNA was extracted from the striatum of all experimental groups using Trizol reagent (ThermoFisher Waltham, MA, USA). mRNA was enriched with magnetic beads with oligo (DT). After adding fragmentation buffer, the first cDNA strand was synthesised with random hexamers using mRNA as a template and the second cDNA strand was synthesised by adding buffer, dNTPs, and DNA polymerase I. The cDNA was purified using AMPure XP beads, and the end product was repaired. The A-tail was added and connected to the sequencing connector, and ampere XP beads screened the fragment size. The cDNA library was constructed by PCR enrichment. The Qubit2.0 was used for preliminary quantitative detection. After diluting the library to 1 ng/UL, the insert size of the library was detected by the Agilent 2,100 system, and the effective concentration of the library was quantified by the real-time quantitative PCR detecting system (the effective concentration of the library was more than 2 nM). Transcriptome analysis (RNA-seq) was performed by Illumina HiSeqTM 2000.

### High throughput sequencing and transcriptomics analysis

The extracted RNA samples were sent to Nuohe Zhiyuan company for high-throughput sequencing and transcriptomics detection. Differently expressed genes in transcriptomic detection were found significant, with a log value greater than 1 or less than −1 and a value of *p* less than 0.05. Significant differentially expressed genes were analysed using the DAVID 6.8 bioinformatics functional tool^1^ (Huang et al., 2009). KEGG pathways (Kyoto Encyclopedia of Gene and Genome) and GO-biological process (Gene Ontology) with FDR < 0.05 were identified and visualised by bubble chart with the ggplot R package.

### Cell culture

SH-SY5Y, U87, THP-1, U937, and Jurkat were obtained from the cell centre of Peking Union Medical College. Neuron cell line SH-SY5Y was cultured in DMEM medium and Sijiqing serum, while glioma cell line U87 was cultured in MEM (Minimum Essential Medium) with imported foetal bovine serum (Gibco, Life Technologies, Carlsbad, CA, USA) and antibiotics. For irradiated cell culture, fresh or conditioned MEM supplemented with 10% heat-inactivated foetal bovine serum (Gibco, Life Technologies, Carlsbad, CA, USA), 100 units/mL penicillin, and 100 μg/mL streptomycin (Beijing Solarbio Science & Technology Co., Beijing, China). The culture was maintained at 37°C in a humidified incubator containing 5% CO_2_.

### Radiation treatment

For irradiation, SH-SY5Y, U87, and their combination (1:1) were seeded at a density of 1.7 × 105 in T25 cell culture flasks. After 12–24 h of incubation, cells were irradiated horizontally with ^12^C^6+^ heavy ions at a dose of 1 Gy, 2 Gy, and 5 Gy (165 MeV/u primary energy; linear energy transfer (LET), 30 KeV/μm; intensity, 0.3–0.5 Gy/min) at HIRFL. After irradiation, the fresh medium was replaced immediately and cultured in 37°C incubators for 24 h, after which a conditioned medium from irradiated cells was used to culture immune cell culture and cell viability assay.

### Cell viability assays

Cell viability was analysed through Cell Titer 96® AQueous One Solution Cell Proliferation Assay as per protocol (Cat. G3581, Promega Corporation, Biotech Co., Ltd.). In brief, 100 μL of conditioned medium was seeded with 6,000 immune cells in each 96-well plate. After 24 h, 20 μL of provided reagents were added to each well, incubated for 2 h at 37°C, and evaluated through a fluorometer at 490 nm. The optical density (OD) was used to analyse cell viability. About six duplications were set.

### Transwell migration assay

The transwell migration assay was conducted to analyse the transmigration of THP-1. Twenty-four Transwell inserts with a pore size of 0.4 μm (Corning, USA) were utilised. After irradiation, 500 μL aliquots of the conditioned medium were added (lower chamber), and 105 THP-1 cells in 200 μL of serum-free RPMI-1640 medium were added (upper chambers) in the Transwell inserts. The cells were cultured for 24 h and washed in PBS twice after the medium was discarded. Cells were fixed in formaldehyde (5 min) and stained with crystal violet. Finally, the membrane was analysed using an Olympus-IX71 light microscope.

### Enzyme-linked immunosorbent assay

Cytokine levels in condition medium-irradiated cell culture were determined using enzyme-linked immunosorbent assay (ELISA) kits according to the manufacturer’s instructions of Global Biotech Co., Ltd. Shanghai, China (#ABIN367992), and other materials were obtained from the Beijing Hankehengyu Bio-Technology Co., Ltd. Beijing, China.

### Statistical analysis

Results were expressed as mean ± standard deviation. The statistical analyses were performed using unpaired two-tailed student’s t-test and two-way ANOVA using GraphPad PRISM 8.0 software. The value of *p* <0.05(*), *p* < 0.01(**), and *p* < 0.001(***) were considered to show statistical significance.

## Results

### Behavioural tests and physiological changes

In the present study, rats from both experimental cohorts were exposed to heavy ion radiation vertically on the backside of the head. The cohort-I groups (G1, G2, and G3) irradiated with 15 Gy of ^12^C^6+^ ion, and cohort II groups (G7, G8, and G9) irradiated with 3.4 Gy of ^56^Fe^26+^ ion radiations were analysed after 1 to 3 months, respectively, such that G1 and G7 after 1 month, G2 and G8 after 2 months, and G3 and G9 after 3 months. At the same time, group (G10) from cohort II irradiated with 8 Gy ^56^Fe^26+^ ion radiation was analysed after 2 months. All changes in the irradiated groups were compared with control groups.

**Figure 1:**
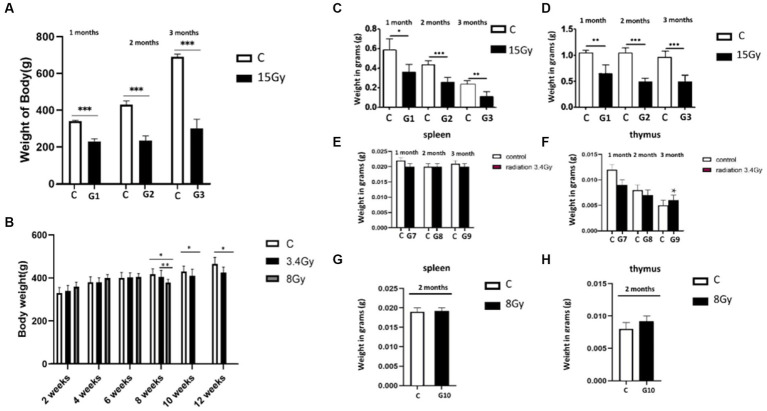
Heavy ion radiation induces neural tissue injury and reduces body weight, thymus, and spleen mass. **(A)** Body weight of cohort I groups (G1–G3, *n* = 6) at 1st, 2nd, and 3rd month after irradiation with ^12^C^6+^ radiations. **(B)** Body weight of cohort II groups (G7–G9, *n* = 6) at 1st, 2nd, and 3rd month, after irradiation with ^56^Fe^26+^ ion radiations. **(C)** Weight of spleen of cohort I groups (G1–G3, *n* = 6) after 1st, 2nd, and 3rd month. **(D)** Weight of thymus of cohort I groups (G1–G3, *n* = 6) after 1st, 2nd, and 3rd month. **(E)** Weight of spleen of cohort II groups (G7–G9, *n* = 6) after 1st, 2nd, and 3rd month. **(F)** Weight of thymus of cohort II groups (G7–G9, *n* = 6) after 1st, 2nd, and 3rd month. **(G)** Weight of spleen of cohort II group (G10, *n* = 6) after 2 months. **(H)** Weight of thymus of cohort II group (G10, *n* = 6) after 2 months. Data are shown as mean + SD in six biological replicates (control) and six biological replicates (radiated groups, G1–G10) **(A–H)** and three technical replicates using an unpaired two-tailed Student’s *t*-test and one-way ANOVA test measured. The differences were considered statistically significant at value of *p*. *p < 0.05, **p < 0.005 vs. control. C, control rats of cohort I and II: G1–G10, group 1–group10.

Initially, physiological changes induced by heavy ion irradiations on the rats were determined by analysing their body weights after exposure to ^12^C^6+^ and ^56^Fe^26+^ ion radiations. Variable physiological changes were observed in both cohorts. Our results showed that all three groups (G1, G2, and G3) of cohort I presented significant weight loss after the 1st, 2nd, and 3rd months than the control groups (Figure 1A). However, cohort II (G7, G8, and G9) presented slow changes in weight loss with ^56^Fe^26+^ ion radiation exposure, such that 3.4 Gy irradiated groups (G7, G8, and G9) did not report significant weight changes till 6 weeks compared to the control group. However, after 8 weeks, 3.4 Gy rats reported significant weight loss (*p* < 0.01) that remained consistent till the 12th week of the experiment than the control group. The G-10 group (8 Gy) initially reported some weight gain for 4 weeks, but prolonged exposure to 8 Gy of ^56^Fe^26+^ ions also resulted in significant weight loss (p < 0.01) compared to the control group (Figure 1B).

Furthermore, the weight of the spleen and thymus of both cohorts were analysed to determine the impact of irradiation on the immune system. It was observed that the weight of the spleen and thymus was significantly reduced in the cohort I group (G1, G2, and G3) than in the control group, as shown in Figures 1C,D. In contrast, cohort II groups (G7, G8, and G9) showed no significant change in spleen mass after the 1st, 2nd, and 3rd month of irradiation, and thymus weight was increased significantly in the 3rd month compared to control groups (Figures 1E,F), pointing hyperfunctioning of the thymus in response to radiation exposure. However, no significant change in thymus and spleen weight was observed after 2 months in the G10 (8 Gy) irradiated group (Figures 1G,H). Altogether, these findings speculate that ^12^C^6+^ irradiation with a high dose (15 Gy) has a more damaging effect, as revealed by the significant loss in the body weights along with the spleen and thymus masses in the G1–G3 groups. However, an increase in the thymus size after 3 months of the ^56^Fe^26+^ small dose (3.4 Gy) might be due to the rebound phenomenon after atrophy caused by radiation exposure, as supported by Moini et al. (2020).

### Heavy ion radiation causes behavioural abnormalities

**Figure 2:**
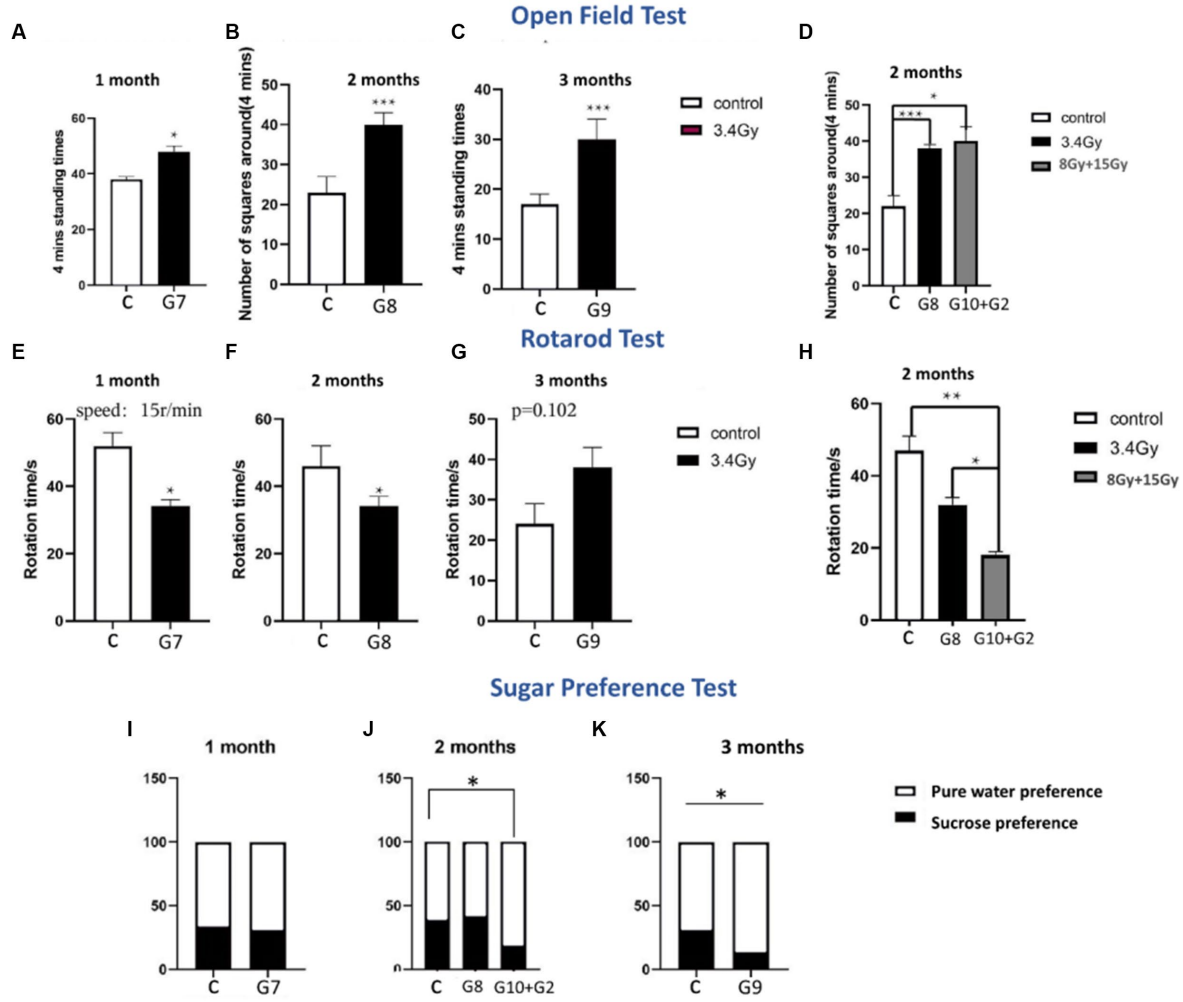
Behavioral tests of both cohorts. **(A–D)** Open field tests of both cohorts (G1–G10, *n* = 6/g). **(E–H)** Rotarod tests of both cohorts (G1–G10, *n* = 6/g). **(I–K)** Sugar preference level of both cohorts (G1–G10, *n* = 6/g). Data are shown as mean + SD in six biological replicates (control) in six biological replicates (radiated groups, G1–G10) and three technical replicates using an unpaired two-tailed Student’s *t*-test and one-way ANOVA test. The differences were considered statistically significant at the value of *p*. *p < 0.05, **p < 0.005 vs. control. C, control rats of cohort I and II: G1–G10, group 1–group10.

Furthermore, to determine the aftereffects of heavy ion radiation on brain functionality, we behaviourally tested rats after 1, 2, and 3 months of exposure to ^12^C^6+^ and ^56^Fe^26+^ ion radiations. Variable behavioural abnormalities were observed in both experimental cohorts. Behavioural tests of both cohorts showed that heavy radiation significantly impacts brain function to impair emotions and trigger long-term cognitive dysfunction. Analysis of sucrose preference tests in both cohorts showed that long-term radiation exposure led to anhedonia, a characteristic feature of depressive behaviour. Up to 2 months, cohort II groups (G7 and G8) did not display significant differences in the sugar and water intake than the control group (Figures 2I,J). However, after the third month of irradiation, cohort II group (G9) showed a significantly lower sugar water intake (*p* < 0.05), as shown in Figure 2K. In line with these findings, the G10 group irradiated with ^56^Fe^26+^ ion (8 Gy) and G2 group irradiated with ^12^C^6+^ (15 Gy) showed a significant loss of interest in sugar water after 2 months than the control group (*p* < 0.05), as shown in Figure 2J, illustrating that high dose irradiations are more lethal and might promote neuronal and neuropsychological issues, compared to low range dose of ^56^Fe^26+^ (3.4 Gy).

The open field test was performed to analyse the anxious behaviour of both irradiated cohorts I and II. Results have shown that the G7, G8, and G9 groups of cohort II (3.4 Gy) developed significant anxiety levels (*p* < 0.05) when exposed to a new environment (Figures 2A–D) in all 3 months, showing more standing time towards the wall and crossing a large number of squares. However, the severity of anxiety increases in the 2nd and 3rd months of irradiation (*p* < 0.05) as illustrated by parameters such as a significantly high number of squares travel time and time facing towards the wall (Figures 2B–D). Moreover, the G10 group (^56^Fe^26+^ ion: 8 Gy) and G2 group (^12^C^6+^: 15 Gy) of both cohorts showed that rats develop anxiousness after 2 months of irradiation (Figure 2D), as demonstrated by the crossing of the significantly large number of squares (*p* < 0.05) compared to the control group, indicating that higher dose and long-term effects of radiations pose deleterious impact on the brain functioning and lead to cognitive issues.

Additionally, rota rod tests are utilised to measure motor coordination ability. Initially, both irradiated cohort’s groups (G7: 3.4 Gy, G10: 8 Gy, and G2:15Gy) showed less physical activity and spent significantly less time on rotating wheels (p < 0.05) than the control group after 1 and 2 months of irradiation (Figures 2E,F,H), indicating that irradiation might affect several brain parts, which leads to coordination disabilities to withstand stress. However, after 3 months of irradiation, the cohort II group, G9 (3.4 Gy), spent more time on rotating wheels than the control group, but no significant difference (*p* = 0.102) was found between the two groups (Figure 2G). Hence, this peculiarity in behaviour reflects that rats might have developed adaptive behaviour towards this stimulus or can be a false negative result that might be influenced by various factors involved during experimentation.

### Heavy ion radiation dysfunction glucose metabolism

**Figure 3:**
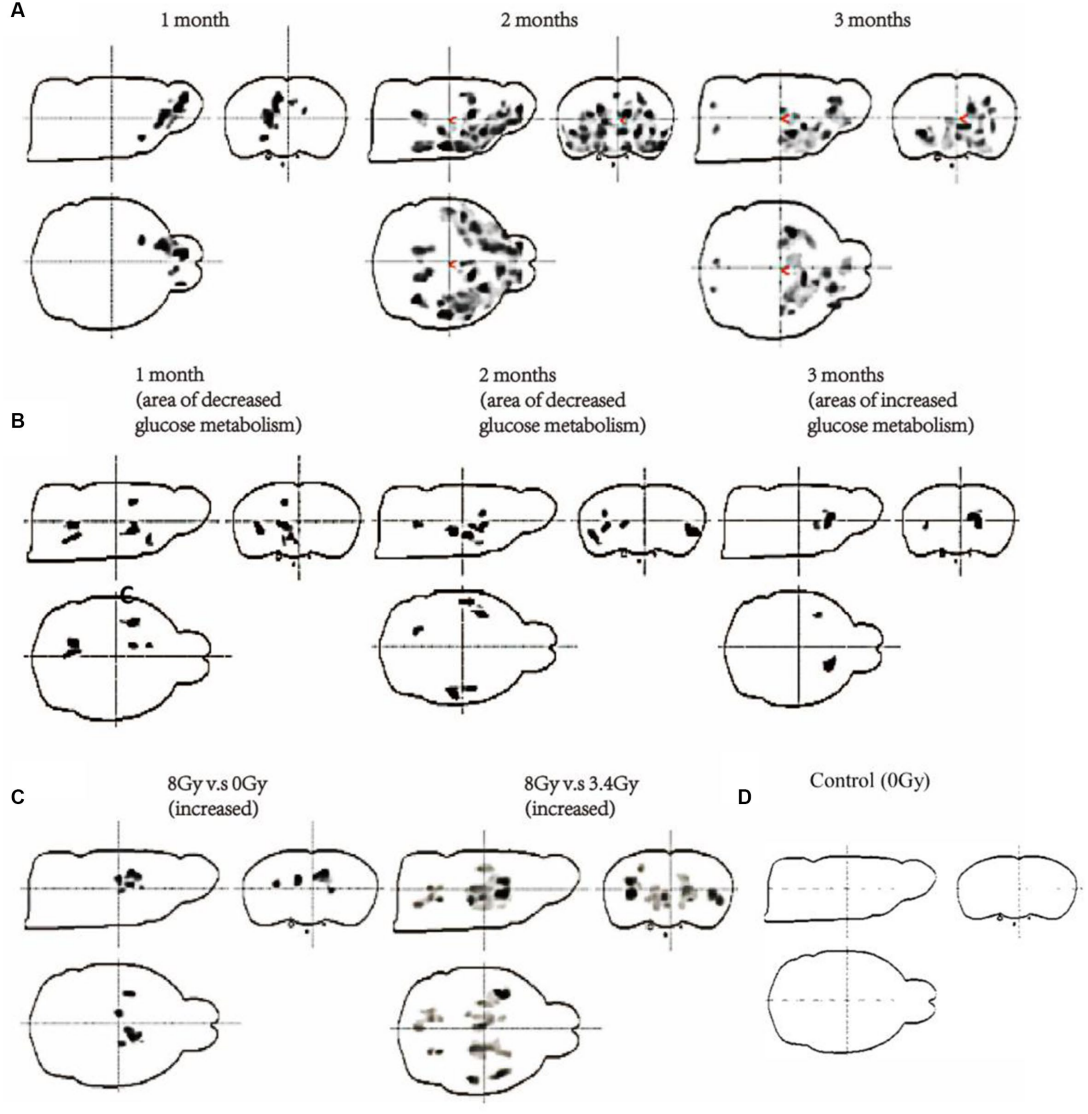
Measurement of glucose metabolism of the brain of both cohorts through ^18^F-FDG-PET scanning. **(A)** Changes in glucose metabolism of cohort I after 15 Gy irradiation with ^^12^C^6+^^ ions for 1, 2, and 3 months (G1–G3, *n* = 6/g). **(B)** Changes in glucose metabolism of cohort II after 3.4 Gy irradiation with ^^56^Fe^26+^^ ions (G7–G9, *n* = 6/g) for 1, 2, and 3 months. **(C)** Comparison of glucose metabolism after 2 months of 8 Gy vs. 0 Gy and 8 Gy vs. 3.4 Gy irradiation with ^^56^Fe^26+^^ ions (G10, *n* = 6/g). **(D)** Control group ^18^F-FDG-PET scan.

Next, the intensity of brain damage caused by different ^56^Fe^26+^ and ^12^C^6+^ irradiations at multiple conditions was investigated by measuring glucose (^18^F-FDG) metabolism in the rat’s brain. Both cohorts were injected with ^18^F-FDG, a glucose analogue that does not undergo glycolysis when taken up by brain cells. The glucose utilisation and cell viability of different brain regions of both cohorts were analysed by ^18^F-FDG-PET scans. In these results, a medium grey/black area shows sites of glucose metabolism and a lighter colour shows more minor changes in glucose metabolism. In contrast, the white area shows no change in glucose metabolism. The results from cohort I showed that the G1 group, after the 1st month of 15Gy ^12^C^6+^ irradiations, presented a significant glucose hypometabolism in the striatum, olfactory sphere, and prefrontal joint cortex region than other parts of the brain displaying glucose hypermetabolism (Figure 3A). However, in G2 group after 2nd month of irradiation, changes in the glucose metabolism increased. Among other brain parts, the amygdala, striatum, and prefrontal cortex presented a significant decline in glucose metabolism (Figure 3A), demonstrating that glucose metabolism was more prominent in the brain’s front side. Final observations obtained from the G3 group after the 3rd month showed that the glucose metabolism rate remained almost the same as in the 2nd month. In contrast, the degree and location of glucose metabolic changes remained slightly altered (Figure 3A). Same brain regions, including the amygdala, striatum, and cortex, displayed glucose hypometabolism. The overall analysis showed that glucose hypometabolism penetrated the front brain regions due to prolonged radiation exposure, especially in the amygdala, striatum, and cortex, reflecting impaired functioning of these particular brain regions, particularly sensory and cognitive processing.

Additionally, the cohort II ^18^F-FDG-PET scans showed dynamic glucose metabolism changes at multiple time zones. After 1st month of 3.4 Gy ^56^Fe^26+^ irradiation in the G7 group, various brain regions, including the striatum, cortex, and hippocampus, displayed significant glucose hypometabolism than other parts of the brain compared with the control group (Figures 3B,C). Among these regions, the striatum showed the most considerable glucose hypometabolism. The G8 group of cohort II revealed that glucose hypometabolism increases most significantly in the striatum region compared to the control group, demonstrating that decreased cellular metabolism might result in impaired striatum functioning (Figure 3B). Alternatively, in the G9 group, ^18^F-FDG-PET scans show glucose hypermetabolism in the striatum region than the control group (Figures 3B,C). However, glucose metabolism of other brain regions did not show any significant difference with the control group.

Interestingly, the analysis of ^18^F-FDG-PET scans of the 8 Gy irradiated group (G10) after 2 months of irradiation has demonstrated glucose hypermetabolism in the hippocampus and striatum region, but most pronounced in the striatum region (Figure 3C). However, this peculiarity in glucose metabolism between 8 Gy and 3.4 Gy ^18^F-FDG-PET scans after 2 months in G8 and G10 showed that 8 Gy has deeply penetrated the brain and resulted in significant neuronal damage, like the findings as obtained from the G9 of 3.4 Gy-^56^Fe^26+^ irradiation (Figures 3C,D). Therefore, based on these findings and previous reports (Onorato et al., 2020; Mancini et al., 2021), it is hypothesised that hypermetabolism in the striatum region after prolonged irradiation might result in neuronal damage, which triggers neuroinflammation by activating microglia and astrocytes and leads to more glucose consumption to repair striatal damage.

Interestingly, the abnormal glucose metabolism in the striatum is also linked with our behaviour test results, showing voluntary movements in open field tests and behaviour in rotarod tests. Hence, based on the persistent changes observed in the striatum region of both cohorts as exposed by behaviour tests and ^18^F-FDG-PET scans, we selected striatum samples of cohort II (G7, G8, G9, and G10 groups, *n* = 3 per group) for the transcriptomic analysis to identify the underlying molecular changes involved in striatum damage and repairment at multiple radiation conditions.

### Heavy ion irradiation triggers an immune effect in the striatum

**Figure 4:**
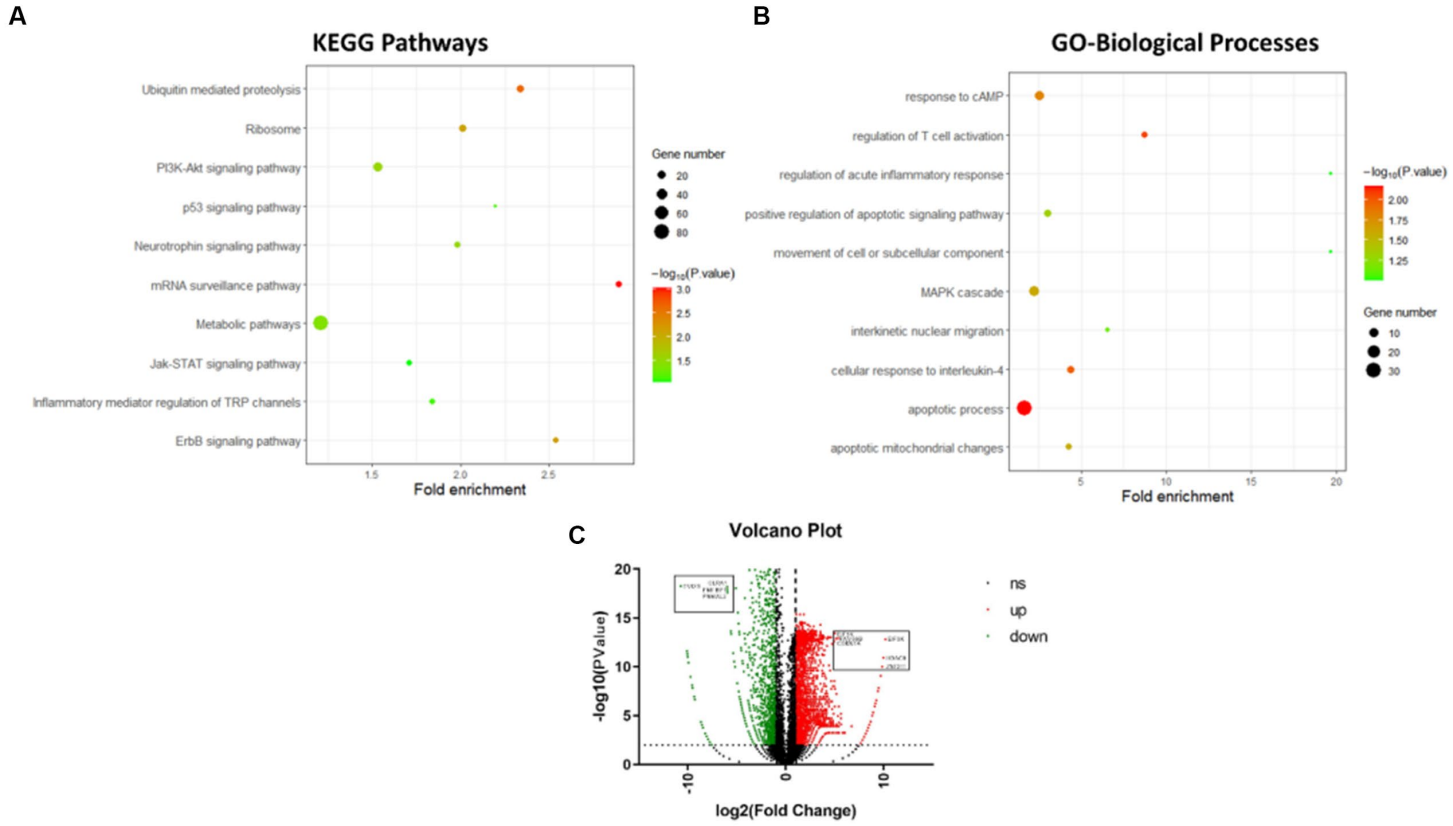
Transcriptomics analysis of differentially expressed genes of striatum after irradiation. **(A)** Top 10 KEGG pathways of differentially expressed genes of striatum after irradiation. **(B)** Top 10 Gene Ontology-biological processes of differentially expressed genes of striatum after irradiation. **(C)** Volcano Graph of differentially expressed genes of striatum after irradiation.

According to the transcriptomic analysis of striatum samples, about 2,402 genes were significantly upregulated, including EIF3K, FAM96B, CDKN1A, HDAC9, and ZNF211, at different radiation conditions. In contrast, 3,172 genes were significantly downregulated, including EVI2B, GLRA1, PMFBP1, and PNMAL2, compared to the control (Figure 4C). The top 10 upregulated signalling pathways involved in regulating the striatum of irradiated cohort II are illustrated in Figure 4A. KEGG analysis of differentially expressed genes among these groups shows that neuroinflammatory pathways are highly active along with metabolic pathways. G7 group (3.4 Gy) shows that the mTOR signalling pathway, neuroactive ligand-receptor interaction pathway, Gap junction pathway, GABAergic synapse pathway, protein digestion and absorption pathway, inflammatory mediator regulation of TRP channels pathway, glutamatergic synapse pathway, calcium signalling pathway, cAMP signalling pathway, and PI3K-Akt signalling pathway as the most stringent pathways. After the 2nd month of irradiation, the G8 group shows a slight change in gene expression regulating antigen processing and presentation, cell adhesion molecules (CAMs) pathway, insulin signalling pathway, cGMP-PKG signalling pathway, calcium signalling pathway, cAMP signalling pathway, PI3K-Akt signalling pathway, mTOR signalling pathway, adipocytokine signalling pathway, toll-like receptor signalling pathway, and melanogenesis pathway. However, the G9 group has demonstrated glucuronic acid conversion, glutamatergic synapses, apoptotic signalling pathways, PI3K-Akt signalling pathway, Jak–STAT signalling pathway, ubiquitin-mediated proteolysis, ErbB signalling pathway, and ribosome as significant pathways than control (Figure 4A).

Similarly, the G10 group’s (8Gy) striatum was found to regulate the mTOR signalling pathway, adipocytokine signalling pathway, HIF-1 signalling pathway, insulin signalling pathway, Jak–STAT signalling pathway, and PI3K-Akt signalling pathway, compared to the control. Interestingly, the PI3K-Akt signalling pathway remains active in all irradiated groups (G7–G10: 3.4 Gy and 8 Gy of ^56^Fe^26+^), whereas the mTOR signalling pathway was observed in an active state for 2 months (G7, G8, and G10). The active phase of the PI3K-Akt signalling pathway and mTOR signalling pathway, in addition to other neuroinflammatory pathways, has depicted the activation of glial cells to repair neuronal damage induced by radiation. However, altered expression of glucose metabolic pathways has also reflected sequel damage or repair of the striatum over variable radiation exposure.

Furthermore, the active biological processes involved in regulating striatum function during different irradiation conditions were analysed through Gene Ontology. The top 10 upregulated biological processes are shown in Figure 4B. The differential gene expression of the G7 group shows the involvement of response to cAMP, anion transport, anion transmembrane transporter activity, apoptotic process, chemical homeostasis, substrate-specific transmembrane transporter activity, and MAPK cascade when compared with control. In the G8 group, other significant biological processes include movement of a cell or subcellular components, regulation of acute inflammatory response, apoptotic mitochondrial changes, MHC protein complex, and antigen processing. In contrast, the G9 group demonstrated regulation of T cell activation, interkinetic nuclear migration, positive regulation of apoptotic inflammatory response, glucocorticoid stimulus, etc. However, based on the dose effects, no significant difference in biological pathways was found between 3.4 Gy and 8 Gy after 2 months. In contrast, a substantial difference in biological pathways was observed between 3.4 Gy and 0 Gy, including the MHC protein complex, antigen processing, and MAPK cascade. Taken together, it can be inferred from the above analysis that as the irradiation dose range increases with time, the level of neuronal damage rises, leading to the activation of neuroinflammatory pathways and other linked pathways, postulating a peculiar cell signalling between neurons and immune cells.

### Heavy ion radiations trigger immune effects in a neural *in vitro* system

**Figure 5:**
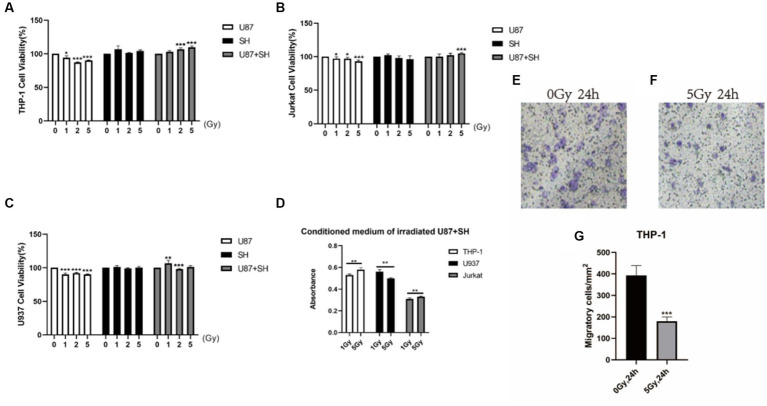
Heavy ion-irradiated neural cells mediate immune effects *in vitro*. **(A)** THP-1 cells (*n* = 3/g), **(B)** U937 cells (*n* = 3/g) were cultured in three different types of conditioned medium, and cell proliferation was determined after 24 h. **(C)** The MTT assay of the conditioned medium after heavy ion irradiation showing different dose-dependent effects on different immune cells. **(D,E)** Representative illustration of THP-1 in transwells (bar = 100 μM) (*n* = 3/g). **(F)** Quantification of THP-1 cell migration (*n* = 3/g). Data are shown as mean + SD in control (three biological replicates), radiated groups (three biological replicates), and three technical replicates using an unpaired two-tailed Student’s *t*-test and one-way ANOVA test. The differences were considered statistically significant at the value of *p*. *p < 0.05, **p < 0.005 vs. control. U87, glial cells: SH, neuronal cell: U87 + SH, glial +neuronal cells: /g, per group.

To study the impact of heavy ion radiations on the cell signalling between neuronal and immune cells, we established brain *in situ* condition by culturing SH-SY5Y cells (neuron-like cells) and U87 cells (astrocytic glial cells) together, following a preliminary study based on primary neurons (see Supplementary File S1), as described previously by our team (Wang et al., 2014) and then irradiated with ^12^C^6+^ ions (1, 2, and 5 Gy). After irradiation, 24 h medium was collected and subjected to immune cells (THP-1: human leukaemia monocytic cell line, Jurkat: human leukemic T-cell line, and U937: human histiocytic lymphoma cell line) as prescribed markers of astrocyte activation. Our results showed that irradiated nerve cells produce differential effects on different immune cells. As shown in Figure 5A, U87 condition medium produced inhibitory effects on THP-1 proliferation at 2 Gy and 5 Gy radiation dosage, while SH condition medium produced no effect on THP-1 cells. However, the conditioned medium of U87 + SH co-culture irradiation has promoted the THP-1 cell viability with increasing radiation dose (2 Gy and 5 Gy). Thus, it speculates that irradiation causes glial cell damage and inhibits the THP-1 viability compared to the control group (0 Gy). In contrast, increased cell viability of THP-1 in the irradiated conditioned medium co-culture U87 + SH demonstrates that signal activation has occurred between neurons and glial cells to phagocytose neuronal debris.

Similarly, low U937 cell viability was observed in the U87 conditional medium after irradiation with 1, 2, and 5 Gy, while negligible radiation damage was observed on U937 cells in the SH medium. On the other hand, co-culture of U87 + SH conditional medium promoted U937 viability at 1 Gy dose; however, a significant decline in U937 cell proliferation was observed at 2 Gy irradiation, following a slight increase in the U937 cell viability with no significant difference at a dose of 5 Gy (Figure 5B), postulating that cytokines release after U937 damage in the nerve cells under co-culture conditions might be influenced by cytokine concentration. Additional investigation on Jurkat cells showed that irradiated U87 condition medium had inhibited Jurkat cell’s activation and proliferation at doses of 1, 2, and 5 Gy doses, whereas a co-cultured, post-radiated U87 + SH conditional medium promoted the activation and proliferation of Jurkat cells at a dose of 5Gy, as observed in our previous findings (Lei et al., 2015). Taken together, these findings indicate that dose-range is strongly linked to the differential expression of different immune cells. For instance, as shown in Figure 5C, THP-1 cell and Jurkat cell activation and proliferation were directly proportional to the increasing dose, whereas U937 cell viability was inversely proportional to the higher dose.

During an active inflammatory response, damaged astrocytes produce cytokines to recruit monocytes (THP-1) from the peripheral immune system and differentiate into macrophages (Chanput et al., 2014). A transwell migration assay was performed to evaluate the ability of these irradiated conditioned medium co-culture glial cells to recruit THP-1 cells and their differentiation into macrophages. Our results showed that a significantly low number of THP-1 cells (*p* < 0.001) migrated in the irradiated conditioned medium compared to the control (Figures 5D–F). Therefore, this indicates that neural cell injury due to irradiations had promoted the viability of the monocytes but reduced monocyte invasion and migration, as previously reported by our research group (Lei et al., 2015).

**Figure 6:**
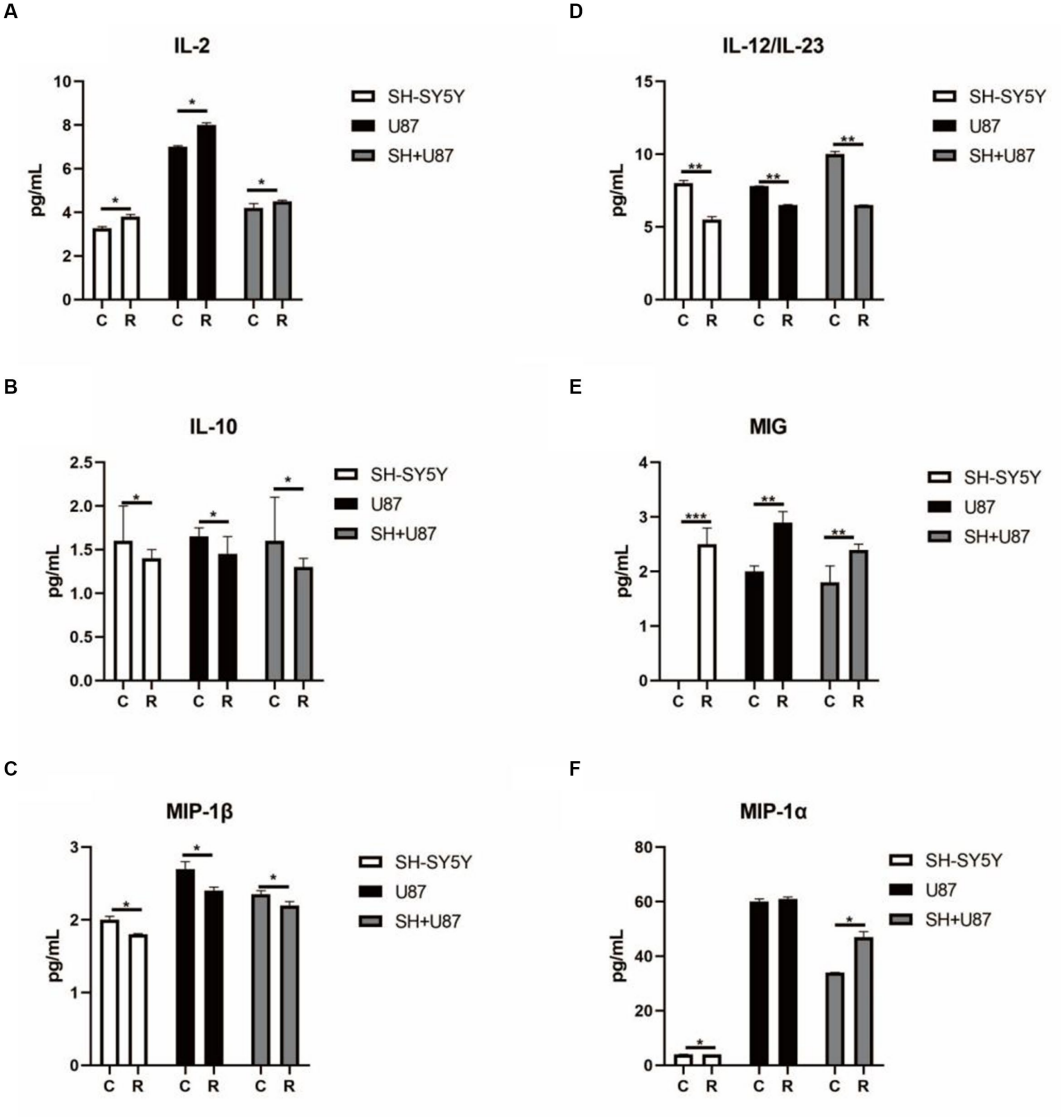
Changes in cytokines linked with immune cell activation in co-culture condition media under heavy ion irradiation. **(A)** The content of IL-2 in different types of condition media (*n* = 3). **(B)** The content of IL-10 in different types of condition media (*n* = 3). **(C)** The content of MIP-1β in different types of condition media (*n* = 3). **(D)** The content of IL-12/IL-23 in different types of condition media (*n* = 3). **(E)** The content of MIG in different types of condition media (*n* = 3). **(F)** The content of MIP-1α in different types of condition media (*n* = 3). Data are shown as mean + SD in three biological replicates (control), three biological replicates (radiated groups), and three technical replicates using an unpaired two-tailed Student’s *t*-test and one-way ANOVA test. The differences were considered statistically significant at the value of *p*. *p < 0.05, **p < 0.005 vs. control. U87, glial cells: SH, neuronal cell: U87 + SH, glial +neuronal cells: /g, per group.

Additionally, to identify the impact of irradiated conditioned medium on immune cells, we also analysed the cytokines levels in the SH-SY5Y cells, U87 cells, and co-culture U87 + SH conditional medium. Interestingly, we observed a significantly higher level of IL-2, MIG, and MIP-1α (*p* < 0.05) in the SH-SY5Y cells, U87 cells, and co-culture U87 + SH conditional medium, indicating that cytokines release had promoted T-cell activation and IFN-γ secretion (Figures 6A,E,F). Similarly, significantly lower levels of IL-10, MIP-1β, and IL-12/IL-23 (Figures 6B–D) in the irradiated SH-SY5Y cells, U87 cells, and co-culture U87 + SH conditional medium (*p* < 0.05) hint at an aggravated immunological reaction in response irradiation. Therefore, based on our results, this complex interplay between neuronal and immune cells in response to heavy ion radiation reflects the dynamic and context-dependent nature of these interactions.

## Discussions

During space expeditions, cosmic radiation and its consequences on the human body are one of the major challenges faced by astronauts. Previous reports have shown that heavy ion radiation causes significant damage to astronauts’ central nervous system, which results in the manifestation of various psychiatric and physiological issues (Onorato et al., 2020). However, the severity of brain damage and underlying molecular mechanisms triggering brain pathologies by heavy ion radiation are still undetermined. This study has designed experiments to investigate the sensitivity of heavy ion radiation on rat brains and associated underlying molecular changes inducing neuronal injuries. Two cohorts of rats were irradiated with ^12^C^6+^ and ^56^Fe^26+^ ion radiation. Each cohort was further subdivided into groups based on dose range and period. Cohort I was exposed to 15 Gy of ^12^C^6+^ ion radiations for 1, 2, and 3 months, whereas cohort II was exposed to ^56^Fe^26+^ ion radiations with 3.4 Gy for 1, 2, and 3 months and 8 Gy for 2 months. Variable dose range and time were used to identify the most sensitive brain regions affected by irradiations. Our results showed that heavy ion irradiation of the brain has persistently affected the functioning of the striatum, which resulted in the abnormal functioning of the immune system.

The striatum controls the movement and reward activities (Hikosaka et al., 2000) and makes neuronal networks with other brain regions, such as the hypothalamus, cortex, and amygdala, to coordinate signal transduction for normal brain functioning (Mello and Villares, 1997; Hollerman et al., 2000). Abnormal functioning of the striatum may result in prominent physiological and behavioural abnormalities (Báez-Mendoza and Schultz, 2013; Mancini et al., 2021). Previous studies on neurodegenerative disorders showed that striatal dopaminergic neuronal degeneration is strongly associated with weight loss and cognitive dysfunction (Contreras-Rodríguez et al., 2017; Pak et al., 2018). Interestingly, our results also supported these manifestations when weight loss was observed in both irradiated cohorts, but the intensity of weight loss was directly proportional to the damage caused by heavy ion radiation. Here, cohort I showed a significant decline in weight due to 15 Gy of ^12^C^6+^ in 3 consecutive months, whereas cohort II showed weight loss after 2 months of 8 Gy and 3 months of 3.4 Gy of ^56^Fe^26+^ ion radiations. Thus, it signifies that high doses of heavy ion radiation are lethal and directly cause cellular damage or apoptosis, even if exposed for a short time. In contrast, lower doses of heavy ion radiation induce structural and functional abnormalities with exposure rate and turn lethal when exposed for a long time.

Similarly, behavioural tests of both cohorts showed that cognition dysfunction increases with radiation exposure. Cohorts I and II exposed to high radiation doses (8 Gy, 15 Gy) showed significant behavioural abnormalities such as anhedonia, low motor coordination, and anxiety levels after 2 months of exposure, whereas cohort II with 3.4 Gy exposure showed significant behavioural abnormalities after 3 months; however, cohort I rats (3.4 Gy) developed more adaptability toward the rotarod test after 3 months. This improved motor coordination behaviour is attributable to multiple reasons. (i) Overweight rats may have sustained rotations due to weight gain. (ii) synaptic plasticity in the striatum and other brain regions makes their motor neurons develop adaptive behaviour to sustain rotations for longer. However, most of these behavioural abnormalities observed in both cohorts are associated with striatum functioning (Isovich et al., 2001). Behaviour deficits are one of the major problems astronauts face, affecting their performance and cosmic radiations are believed to be one culprit (Parihar et al., 2016; Constanzo et al., 2020). Still, underlying neurological changes have not yet been studied due to limited research. Therefore, in line with our results, it can be speculated that the striatum, whose dysfunction resulted in long-term cognitive dysfunction, is more vulnerable to radiation than other organs.

Furthermore, the underlying brain damage of both cohorts was executed by measuring glucose metabolism through ^18^F-FDG-PET scans. Our results showed persistent glucose hypometabolism in the striatum in cohort I (15 Gy) after 3 months of irradiation. In contrast, cohort II (3.4 Gy) showed hypometabolism in the striatum for 2 successive months after irradiation. Glucose hypometabolism in brain disorders is associated with microstructural abnormalities, and many neurodegenerative disorders exhibit glucose hypometabolism in different brain regions (Samuraki et al., 2015). Thus, reduced ^18^F-FDG uptake by the striatum region in our results implies oxygen deprivation, neuronal injury, or cell death (Hamilton et al., 2018; Han et al., 2021), which might be due to high dose irradiation in cohort I and initial cellular damages in cohort II. Interestingly, increased glucose uptake was observed in cohort II groups irradiated with 8 Gy (2 months) and 3.4 Gy (3 months). Several studies reported that during brain insults, inflammatory cells infiltrate tissue with limited blood supply to the damaged neurons and astrocytes due to a lack of oxygen and glucose availability. During this process, inflammatory cells metabolise glucose non-oxidatively to meet their energy demands, raising glucose levels to normal levels and often masking metabolic deficits in various neurogenerative diseases (Backes et al., 2016). Hence, it supports that hypermetabolism in the cohort II group might result from activating inflammatory cells.

Based on the findings in cohort II as observed by physiological, behavioural, and ^18^F-FDG-PET scanning and intensity of LETs causing striatum dysfunction. We selected cohort II for transcriptomics analysis. Our findings showed the activated neuroinflammatory pathways in addition to glucose metabolism pathways. Among various pathways, the PI3K-Akt signalling pathway remains active in all irradiated groups, while the mTOR signalling pathway was observed in an active state for 2 months. Various studies reported that activated pro-inflammatory cytokines amplified PI3K-mTOR signalling in the brain region during brain injury and resulted in chronic neuroinflammation and pain (Rai et al., 2019; Zhang et al., 2019). Furthermore, it is proposed that abnormal immune activation in the striatum and mid-brain can be a major factor in inducing neurodegeneration by mediating cell-to-cell death signals or toxicity triggered by soluble pro-inflammatory cytokines (Klawonn et al., 2021; Mancini et al., 2021). Hence, in line with previous studies, the impaired immune system by radiation was also analysed by measuring spleen and thymus health. We found a significant decline in thymus and spleen mass in cohort I, while cohort II presented low mass thymus after 3 months of 3.4 Gy and 2 months of 8 Gy irradiation.

Furthermore, to intervene, insights into the molecular responses between neurons and immune cells in response to heavy ion radiation causing striatum dysfunction, we established an *in situ* brain-like environment by co-culturing SH-SY5Y and U87 cells followed by irradiation with ^12^C^6+^ ions at various doses and identified the impact of irradiation on cell signaling cytokine levels and immune cell behaviour. Our findings showed that irradiated nerve cells have differential effects on different immune cells, underscoring the complexity of interactions between these cell types. For instance, here in our study, the inhibitory effects of the U87-conditioned medium on THP-1 proliferation, particularly at 2 and 5 Gy radiation doses, suggest that irradiation may cause damage to glial cells. Glial cells are known to play crucial roles in supporting and protecting neurons, and their damage can influence immune cell viability (Devanney et al., 2020). Accumulating evidence has reported that impaired astrocytes produce cytokines to recruit monocytes (THP-1) from the peripheral immune system and differentiate them into macrophages (Chanput et al., 2014; Devanney et al., 2020). Interestingly, the conditioned medium U87 + SH co-culture irradiation-promoted THP-1 cell viability with increasing doses. Hence, this suggests that irradiation may lead to glial cell damage and impaired THP-1 viability, which might be rescued through signalling interactions between neurons and glial cells for phagocytosis of neuronal debris, coherent with our team’s previous findings (Lei et al., 2015). Similarly, low U937 cell viability in the U87-conditioned medium is affected by cytokine release after U937 cell damage. However, co-culture U87 + SH-conditioned medium promoted U937 viability at low radiation dose, while at 2 Gy irradiation, there was a significant decline in U937 cell proliferation and recovered U937 cell proliferation again at 5 Gy irradiation, suggesting that this shift of U937 cell proliferation might be influenced by cytokine concentration and the microenvironment-regulating immune cell responses. In-line with these results, our previous findings (Lei et al., 2015) using Jurkat cells also revealed that irradiated U87-conditioned medium inhibited Jurkat cell activation and proliferation at various radiation doses, while U87 + SH-conditioned medium promoted the activation and proliferation of Jurkat cells at higher doses. Moreover, these findings also emphasise the dose-dependency of immune cell responses to radiation, where activation and proliferation of THP-1 cells and Jurkat cells are directly proportional to increasing radiation dose, while U937 cell viability is inversely proportional to the dose. Thus, it highlights the complexity of immune responses in the presence of radiation and suggests that different immune cells may be influenced differently by radiation doses. In support of these findings, Parente et al. (2020) also reported glial activation in rats in response to whole-brain irradiation; however, the interplay of these glial cells with neurons is still under investigation (Smith et al., 2012). Therefore, here, we highlight the multifaceted interactions between neuronal and immune cells in response to heavy ion radiation and provide valuable insights into the complex mechanisms governing radiation-induced immune responses, with potential implications for the development of strategies to overcome the radiation hazards and enhancing the efficacy of radiation therapy for various ailments.

Moreover, we also observed the changes in the cytokines and their role in immune activation to understand the interplay between irradiated neuronal and glial cells and the immune response. Cytokines are signalling molecules that play a pivotal role in regulating various aspects of the immune system, including immune cell activation, differentiation, and proliferation (Parente et al., 2020). Notably, this study found significantly higher levels of IL-2, MIG, and MIP-1α, while significantly lower levels of IL-10, MIP-1β, and IL-12/IL-23 were observed in the irradiated SH-SY5Y cells, U87 cells, and co-culture U87 + SH-conditioned medium. IL-2 is a key cytokine that plays a crucial role in the activation and proliferation of T-cells. It acts as a T-cell growth factor, stimulating the expansion of T-cell populations. Several studies have reported that increased levels of IL-2 play a prominent role in exacerbating neurodegenerative processes in various neurological disorders such as Alzheimer’s disease (Ribas et al., 2002; Stessin and Ryu, 2017). Hence, higher levels of IL-2 in response to irradiated conditioned medium suggest an activation of T-cells, resulting in neuroinflammation. Similarly, MIG and MIP-1α are chemokines induced by gamma interferon (IFN-γ) and involved in attracting T-cells to sites of inflammation. Higher levels of MIG and MIP-1α in our study have confirmed the evidence reported by several studies illustrating that MIG and MIP-1α recruit immune cells and contribute to the neuroinflammatory response to brain damage in various neurological disorders such as multiple sclerosis (Rydbirk et al., 2019). However, the reduction of the IL-10, MIP-1β, and IL-12/IL-23 in irradiated condition medium is noteworthy as IL-10 and IL-12/IL-23 are anti-inflammatory cytokines that play significant roles in preventing inflammatory cascades and associated pathologies. According to our findings and in support of previous reports, it is inferred that the lower expression of IL-10 and IL-12/IL-23 may aggravate the pathological conditions and neuronal damage (Miñano et al., 1996; Carlini et al., 2023). MIP-1β (macrophage inflammatory protein-1beta) is another cytokine that plays a crucial role in immune cell recruitment. Previous reports have shown that MIP-1β is produced in response to brain damage and activates immunological cells (neutrophils, microglia, and astroglia) to induce neuroinflammatory processes in neurological disorders (Howes et al., 2014). On the other hand, MIP-1β knockdown displays therapeutic properties and results in motor recovery accompanied by lowering neuroinflammatory responses (Thompson et al., 2008). Hence, in this notion, lowered levels of MIP-1β found in our study may be an adaptive response to compensate for the neuronal damage caused by irradiation by interfering with immune cell trafficking and activation. In line with our findings, several studies also reported changes in the inflammatory environment in the brains of the irradiated rats. For example, Lee et al. (2010) reported that radiation resulted in the significantly upregulated expression of TNF-α, IL-1β, and MCP-1 in the hippocampus and cortex region of the irradiated rats and mouse BV-2 microglial cells (Lee et al., 2010). Similarly, an elevated level of TNF-α and microglial migration in the hippocampus of irradiated mice was reported to play a prominent role in radiation-induced neurocognitive toxicity (Chu et al., 2020). Taken together, changes in the pro-inflammatory cytokines suggest a complex and dynamic response of the immune system to irradiated neuronal and glial cells. These changes marked by elevated IL-2, MIG, and MIP-1 MIP-1α and decreased IL-10, MIP-1β, and IL-12/IL-23 suggest a pro-inflammatory microenvironment conducive to neuroinflammatory processes. Such insights into cytokine modulation post-irradiation not only illuminate potential risks for neurological conditions in space missions but also present opportunities for therapeutic intervention. Therefore, it is suggested that understanding and manipulating these cytokine dynamics may hold a promise in devising strategies aimed at mitigating inflammation-associated pathologies, thereby paving the way for more effective preventive and therapeutic measures in space exploration and neurological disorders.

## Conclusion

In summary, we investigated the damaging intensity of the brain under heavy ion radiation. This study was performed in two parallel cohorts exposed to different radiations in multiple doses and times. Cohort I was irradiated with 15 Gy in three groups for 1, 2, and 3 months, whereas cohort II was irradiated with 3.4 Gy (three groups for 1, 2, and 3 months) and 8 Gy (one group for 2 months) with ^56^Fe^26+^ irradiation. Results showed that these irradiations have resulted in striatum dysfunction in both cohorts as revealed by physiological, behavioural, ^18^F-FDG-PET scans. Transcriptomic analysis showed that striatum dysfunction is linked with an abnormal immune system. *In vitro* ^12^C^6+^ ions studies showed that irradiation induces different effects on different immune cells and allows monocyte proliferation but inhibits its differentiation and migration, resulting in chronic neuroinflammation in the striatum and might affect other associated brain regions, resulting in cognitive deficits and other pathological manifestations. Overall, these intricate findings underscore the importance of elucidating the underlying molecular mechanisms governing these responses and point to potential therapeutic strategies for mitigating the adverse effects of heavy ion radiation exposure. This research significantly advances our understanding of heavy ion radiation’s intricate consequences, offering a robust foundation for future investigations in the field of radiation biology and space exploration.

## Data availability statement

The original contributions presented in the study are included in the article/Supplementary material, further inquiries can be directed to the corresponding author.

## Ethics statement

Ethical approval was not required for the studies on humans in accordance with the local legislation and institutional requirements because only commercially available established cell lines were used. The animal study was approved by Ethics committee of Beijing Institute of Technology, China. The study was conducted in accordance with the local legislation and institutional requirements.

## Author contributions

ZC and HM contributed to the study design, experimental work, funding, and writing manuscript. YL, MR, and HW performed the software assistance and participated in some experiments and manuscript writing. RL and TZ participated in the implementation of some experiments and visualisation. YD and HM supervised and reviewed the manuscript. All authors contributed to the article and approved the submitted version.

## Footnotes

^1^https://david.ncifcrf.gov/
