# Corrigendum: Altered expression of inflammation-associated molecules in striatum: an implication for sensitivity to heavy ion radiations

**DOI:** 10.3389/fncel.2024.1356536

**Published:** 2024-02-19

**Authors:** Zixuan Chen, Yumeng Li, Madiha Rasheed, Hao Wang, Runhong Lei, Tuo Zhao, Yulin Deng, Hong Ma

**Affiliations:** ^1^Beijing Key Laboratory for Separation and Analysis in Biomedicine and Pharmaceuticals, School of Medical Technology, Beijing Institute of Technology, Beijing, China; ^2^Department of Radiation Oncology, Peking University Third Hospital, Beijing, China

**Keywords:** heavy ion radiations, striatum, immune system, neuroinflammation, astrocytes

In the published article, Moini et al., 2020 was not cited in the manuscript text, or included in the reference list. The reference has been added, and the citation has been inserted in **Results**, *Behavioral tests and physiological changes*, paragraph 3 and should read as follows:

“Furthermore, the weight of the spleen and thymus of both cohorts were analysed to determine the impact of irradiation on the immune system. It was observed that the weight of the spleen and thymus was significantly reduced in the cohort I group (G1, G2, and G3) than in the control group, as shown in Figures 1C, D. In contrast, cohort II groups (G7, G8, and G9) showed no significant change in spleen mass after the 1st, 2nd, and 3rd month of irradiation, and thymus weight was increased significantly in the 3rd month compared to control groups (Figures 1E, F), pointing hyperfunctioning of the thymus in response to radiation exposure. However, no significant change in thymus and spleen weight was observed after 2 months in the G10 (8Gy) irradiated group (Figures 1G, H). Altogether, these findings speculate that ^12^C^6+^ irradiation with a high dose (15Gy) has a more damaging effect, as revealed by the significant loss in the body weights along with the spleen and thymus masses in the G1-G3 groups. However, an increase in the thymus size after 3 months of the ^56^Fe^26+^ small dose (3.4 Gy) might be due to the rebound phenomenon after atrophy caused by radiation exposure, as supported by Moini et al., 2020.”

Additionally, in the published article, Onorato et al., 2020; Mancini et al., 2021 were not cited in the manuscript text. The citation has now been inserted in **Results**, *Heavy ion radiation dysfunction glucose metabolism*, Paragraph 3 and should read as follows:

“Interestingly, the analysis of 18F-FDG-PET scans of the 8 Gy irradiated group (G10) after 2 months of irradiation has demonstrated glucose hypermetabolism in the hippocampus and striatum region, but most pronounced in the striatum region (Figure 3C). However, this peculiarity in glucose metabolism between 8 Gy and 3.4 Gy ^18^F-FDG-PET scans after 2 months in G8 and G10 showed that 8 Gy has deeply penetrated the brain and resulted in significant neuronal damage, like the findings as obtained from the G9 of 3.4Gy-^56^Fe^26+^ irradiation (Figures 3C, D). Therefore, based on these findings and previous reports (Onorato et al., 2020; Mancini et al., 2021), it is hypothesised that hypermetabolism in the striatum region after prolonged irradiation might result in neuronal damage, which triggers neuroinflammation by activating microglia and astrocytes and leads to more glucose consumption to repair striatal damage.”

In the published article, there was an error in the **Funding** statement as some supporting funding information was missing. The correct **Funding** statement appears below.

“This study was supported by the National Natural Science Foundation of China [Grant No. 81601114], Space Medical Experiment Project of China Manned Space Program (No. HYZHXM02003), and Excellent Young Scholars Research Fund of Beijing Institute of Technology.”

In the published article, there was an error in the Supplementary Figure 1 legend. A spelling error was present in Figure 1C, where “primary microgels” should have been written as “primary microglial cells”. The correct statement appears below.

“Supplementary Figure 1. Effect of the survival rate of primary neurons and the migration of primary microglial cells (Transwell) by γ rays (15 Gy). A: Rat primary neurons for 24 hours and 48 hours of irradiation; B: Survival rate of rat primary neurons for 24 and 48 hours of irradiation; C: Primary microglial cells after damage to primary neurons after irradiation Plasma cell migration. Ruler = 100 μm. Compared with the corresponding control group, ^**^ represents p < 0.01, and ^***^ represents p < 0.001.”

In the published article, there was an error in the text. Only the spleen and thymus were weighed during experimentation; therefore, the sentences have been revised for clarification. A correction has been made to **Materials and methods**, *Animal sample collection*, Paragraph 1. The sentence previously stated the following:

“Each cohort of rats was weighed, deeply anaesthetised with pentobarbital sodium (60 mg/kg of body weight, concentration, 20 mg/mL), and then sacrificed. After perfusion with cold saline solution, the striatum, thymus, and spleen were excised on the ice-cold plate, weighed, and washed with phosphate buffer solution, and stored at −80°C for further experimentation.”

The corrected sentence appears below as follows:

“Each cohort of rats was weighed, deeply anesthetized with pentobarbital sodium (60 mg/kg of body weight, concentration: 20 mg/mL), and then sacrificed. After perfusion with cold saline solution, the striatum, thymus, and spleen were excised on an ice-cold plate then washed with phosphate buffer solution and stored at −80 °C for further experimentation. Additionally, thymus, and spleen were weighed before being preserved at −80 °C.”

In the published article, there was an error in the text. Based on recent modifications to Figure 5, in-text figure numbers have been revised and sentences have been modified for clarification.

A correction has been made to the **Results** section, *Heavy ion radiations trigger immune effects in a neural in-vitro system, paragraphs 2 and 3*. This sentence previously stated the following:

“Similarly, low U937 cell viability was observed in the U87 conditional medium after irradiation with 1, 2, and 5 Gy, while negligible radiation damage was observed on U937 cells in the SH medium. On the other hand, co-culture of U87 + SH conditional medium has promoted U937 viability at 1 Gy dose; however, a significant decline in U937 cell proliferation was observed at 2 Gy irradiation, following slight increase in the U937 cell viability with no significant difference at 5 Gy dose (Figure 5C), postulating that cytokines release after U937 damage in the nerve cells under co-culture conditions might be influenced by cytokine concentration. Further investigation on Jurkat cells showed that irradiated U87 condition medium had inhibited Jurkat cell's activation and proliferation at 1, 2, and 5 Gy doses. Whereas a co-cultured, post-radiated U87 + SH conditional medium has promoted the activation and proliferation of Jurkat cells at 5 Gy dose (Figure 5B). Taken together, these findings indicate that dose range is strongly linked to the differential expression of different immune cells. For instance, as shown in Figure 5D, THP-1 cells and Jurkat cell's activation and proliferation were directly proportional to the increasing dose, whereas U937 cell viability was inversely proportional to the higher dose.

During an active inflammatory response, damaged astrocytes produce cytokines to recruit monocytes (THP-1) from the peripheral immune system and differentiate into macrophages (Chanput et al., 2014). A transwell migration assay was performed to evaluate the ability of these irradiated conditioned medium co-culture glial cells to recruit THP-1 cells and their differentiation into macrophages. Our results showed that a significantly low number of THP-1 cells (*p* < 0.001) migrated in the irradiated conditioned medium compared to the control (Figures 5E–G). Therefore, this indicates that neural cell injury due to irradiations had promoted the viability of the monocytes and peripheral immune T cells but reduced monocyte invasion and migration, as previously reported by our research group (Lei et al., 2015).”

The corrected sentence appears below as follows:

“Similarly, low U937 cell viability was observed in the U87 conditional medium after irradiation with 1, 2, and 5Gy, while negligible radiation damage was observed on U937 cells in the SH medium. On the other hand, co-culture of U87+SH conditional medium promoted U937 viability at 1Gy dose; however, a significant decline in U937 cell proliferation was observed at 2Gy irradiation, following a slight increase in the U937 cell viability with no significant difference at a dose of 5Gy (Figure 5B), postulating that cytokines release after U937 damage in the nerve cells under co-culture conditions might be influenced by cytokine concentration. Additional investigation on Jurkat cells showed that irradiated U87 condition medium had inhibited Jurkat cell's activation and proliferation at doses of 1, 2, and 5Gy doses, whereas a co-cultured, post-radiated U87+SH conditional medium promoted the activation and proliferation of Jurkat cells at a dose of 5Gy, as observed in our previous findings (Lei et al., 2015). Taken together, these findings indicate that dose-range is strongly linked to the differential expression of different immune cells. For instance, as shown in Figure 5C, THP-1 cell and Jurkat cell activation and proliferation were directly proportional to the increasing dose, whereas U937 cell viability was inversely proportional to the higher dose.

During an active inflammatory response, damaged astrocytes produce cytokines to recruit monocytes (THP-1) from the peripheral immune system and differentiate into macrophages (Chanput et al., 2014). A transwell migration assay was performed to evaluate the ability of these irradiated conditioned medium co-culture glial cells to recruit THP-1 cells and their differentiation into macrophages. Our results showed that a significantly low number of THP-1 cells (*P* < 0.001) migrated in the irradiated conditioned medium compared to the control (Figures 5D–F). Therefore, this indicates that neural cell injury due to irradiations had promoted the viability of the monocytes but reduced monocyte invasion and migration, as previously reported by our research group (Lei et al., 2015).”

In the published article, there was an error in the text.

A correction has been made to the **Discussions** section, *Paragraph 3*. This sentence previously stated the following:

“Similarly, behavioural tests of both cohorts showed that cognition dysfunction increases with radiation exposure. Cohorts I and II exposed to high radiation doses (8 Gy and 15 Gy) showed significant behavioural abnormalities such as anhedonia, low motor coordination, and anxiety levels after 2 months of exposure, whereas cohort II with 3.4 Gy exposure showed significant behavioural abnormalities after 3 months: however, cohort I rats (3.4 Gy) developed more adaptability towards rotarod test after 3 months. This improved motor coordination behaviour is subjected to multiple reasons: (i) Overweight rats may have sustained rotations due to weight gain; (ii) synaptic plasticity in the striatum and other brain regions makes their motor neurons develop adaptive behaviour to sustain rotations for more time; and (iii) experimental error giving false negative results influenced by various factors. However, most of these behavioural abnormalities observed in both cohorts are associated with striatum functioning (Isovich et al., 2001). Behaviour deficits are one of the major problems astronauts face, affecting their performances, and cosmic radiations are believed to be one culprit (Parihar et al., 2016; Constanzo et al., 2020). Still, underlying neurological changes have not been studied so far due to limited research. Therefore, in line with our results, it can be speculated that the striatum is more vulnerable to radiation than other organs, whose dysfunction resulted in long-term cognitive dysfunction”

The corrected sentence appears below as follows:

“Similarly, behavioural tests of both cohorts showed that cognition dysfunction increases with radiation exposure. Cohorts I and II exposed to high radiation doses (8Gy, 15Gy) showed significant behavioural abnormalities such as anhedonia, low motor coordination, and anxiety levels after 2 months of exposure, whereas cohort II with 3.4Gy exposure showed significant behavioural abnormalities after 3 months; however, cohort I rats (3.4 Gy) developed more adaptability toward the rotarod test after 3 months. This improved motor coordination behaviour is attributable to multiple reasons. (i) Overweight rats may have sustained rotations due to weight gain. (ii) synaptic plasticity in the striatum and other brain regions makes their motor neurons develop adaptive behaviour to sustain rotations for longer. However, most of these behavioural abnormalities observed in both cohorts are associated with striatum functioning (Isovich et al., 2001). Behaviour deficits are one of the major problems astronauts face, affecting their performance and cosmic radiations are believed to be one culprit (Parihar et al., 2016; Constanzo et al., 2020). Still, underlying neurological changes have not yet been studied due to limited research. Therefore, in line with our results, it can be speculated that the striatum, whose dysfunction resulted in long-term cognitive dysfunction, is more vulnerable to radiation than other organs.”

In the published article, there was an error in the text. The first two sentences of this paragraph are inter-linked but written separately, which may cause confusion. Both sentences have been combined into a single sentence.

A correction has been made to the **Discussion** section, Paragraph 6. This sentence previously stated the following:

“Furthermore, to intervene the insights into the molecular responses between neurons and immune cells in response to heavy ion radiation causing striatum dysfunction. We established an in-situ brain-like environment by co-culturing SH-SY5Y and U87 cells followed by irradiation with ^12^C^6+^ ions at various doses and identified the impact of irradiation on cell signalling cytokine levels and immune cell behaviour.”

The corrected sentence appears below as follows:

“Furthermore, to intervene, insights into the molecular responses between neurons and immune cells in response to heavy ion radiation causing striatum dysfunction, we established an *in-situ* brain-like environment by co-culturing SH-SY5Y and U87 cells followed by irradiation with ^12^C^6+^ ions at various doses and identified the impact of irradiation on cell signaling cytokine levels and immune cell behaviour.”

In the published article, there was an error in the text. Based on the results of the updated version of Figure 5, **Discussions** the description of Figure 5 has been modified accordingly.

A correction has been made to **Discussions**, paragraph 6. This sentence was previously stated the following

“Additional investigations to support our finding using Jurkat cells revealed that irradiated U87-conditioned medium has inhibited Jurkat cell activation and proliferation at various radiation doses, while U87 + SH-conditioned medium has promoted the activation and proliferation of Jurkat cells at higher doses.”

The corrected sentence appears below as follows:

“In-line with these results, our previous findings (Lei et al., 2015) using Jurkat cells also revealed that irradiated U87-conditioned medium inhibited Jurkat cell activation and proliferation at various radiation doses, while U87+SH-conditioned medium promoted the activation and proliferation of Jurkat cells at higher doses.”

In the published article, there was an error in the text. The last lines of **Discussions**, paragraph 7, was confusing; therefore we have revised and modified the last line of paragraph 7 for clarity. A correction has been made to **Discussions**, Paragraph 7, Last lines. The sentence previously stated the following:

“These changes point to the possibility of fostering T-cell activation, recruitment, and the development of a pro-inflammatory microenvironment that may have implications in future space missions and also for optimizing therapies that harness radiation-induced immune activation.”

The corrected sentence appears below as follows:

“These changes marked by elevated IL-2, MIG, and MIP-1 MIP-1α and decreased IL-10, MIP-1β, and IL-12/IL-23 suggest a pro-inflammatory microenvironment conducive to neuroinflammatory processes. Such insights into cytokine modulation post-irradiation not only illuminate potential risks for neurological conditions in space missions but also present opportunities for therapeutic intervention. Therefore, it is suggested that understanding and manipulating these cytokine dynamics may hold a promise in devising strategies aimed at mitigating inflammation-associated pathologies, thereby paving the way for more effective preventive and therapeutic measures in space exploration and neurological disorders.”

In the published article, there was an error in Figure 1 and Figure 5 as published. The previous data of Figure 1C, contained a low number of animals, i.e., *n* = 4. We reanalysed the data using six animals in each group and updated Figures 1C, D in Figure 1. The results of Figure 5B were aligned with our previous findings, so we removed them from Figure 5 and mentioned them in the text with a relevant description. Additionally, Figures 5D–F has been updated with the latest images of transwell assay for clarification. The corrected Figure 1 and Figure 5 and their captions appear below.

**Figure 1:**
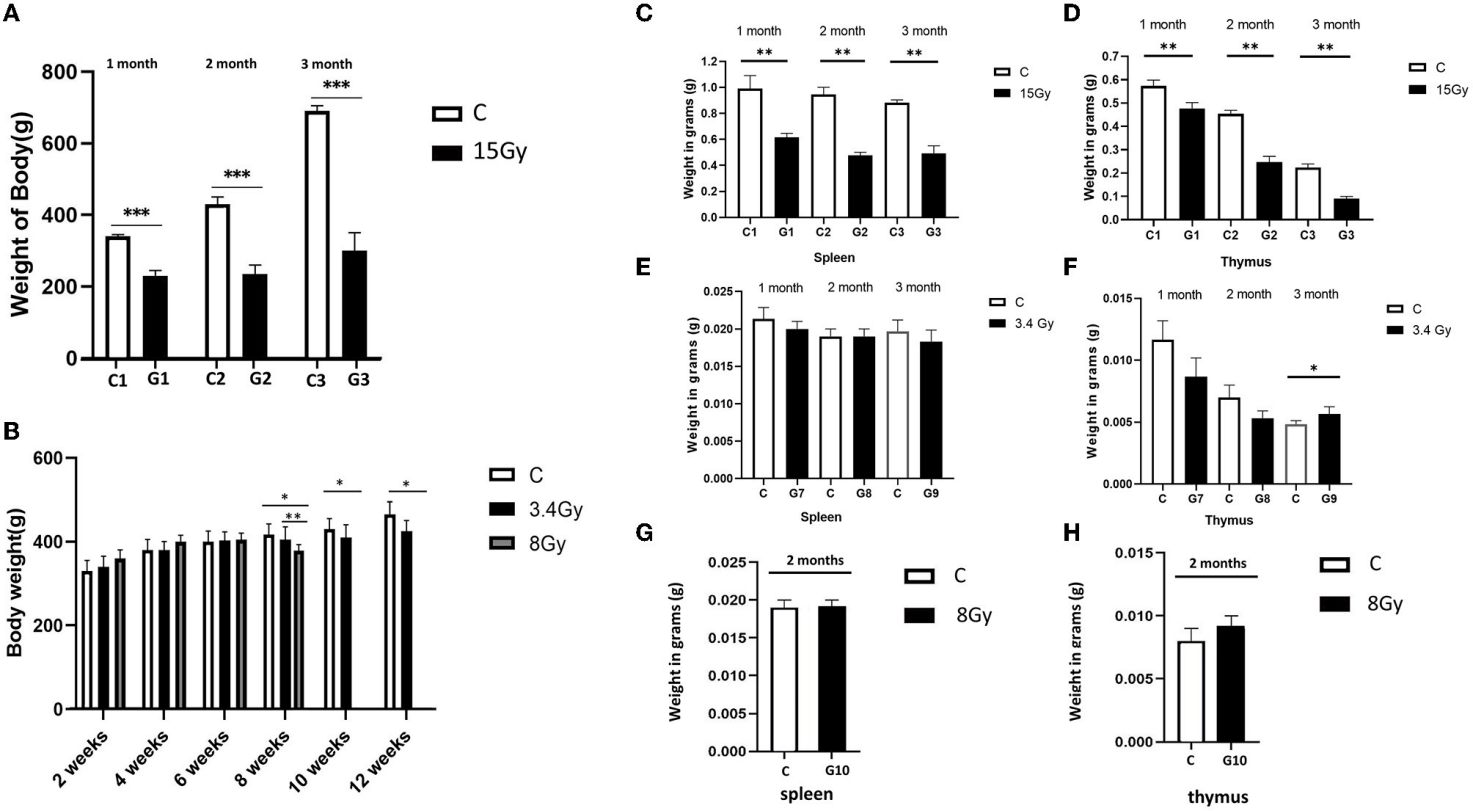
Heavy ion radiation induces neural tissue injury and reduces body weight, thymus, and spleen mass. **(A)** Body weight of cohort I groups (G1–G3, *n* = 6) at 1st, 2nd, and 3rd month after irradiation with ^12^C^6+^ radiations. **(B)** Body weight of cohort II groups (G7-G9, *n* = 6) at 1st, 2nd, and 3rd month, after irradiation with ^56^Fe^26+^ ion radiations. **(C)** Weight of spleen of cohort I groups (G1–G3, *n* = 6) after 1st, 2nd, and 3rd month **(D)** Weight of thymus of cohort I groups (G1-G3, *n* = 6) after 1st, 2nd, and 3rd month **(E)** Weight of spleen of cohort II groups (G7-G9, *n* = 6) after 1st, 2nd, and 3rd month **(F)** Weight of thymus of cohort II groups (G7–G9, *n* = 6) after 1st, 2nd and 3rd month. **(G)** Weight of spleen of cohort II group (G10, *n* = 6) after 2 months **(H)** Weight of thymus of cohort II group (G10, *n* = 6) after 2 months. Data are shown as mean + SD in six biological replicates (control) and 6 biological replicates (Radiated groups, G1–G10) **(A–H)** and three technical replicates using an unpaired two-tailed Student's *t*-test and one-way ANOVA test measured. The differences were considered statistically significant at p-value. ^*^p < 0.05, ^**^p < 0.005 vs control. C, control rats of cohort I and II: G1–G10, group 1–group10.

**Figure 5:**
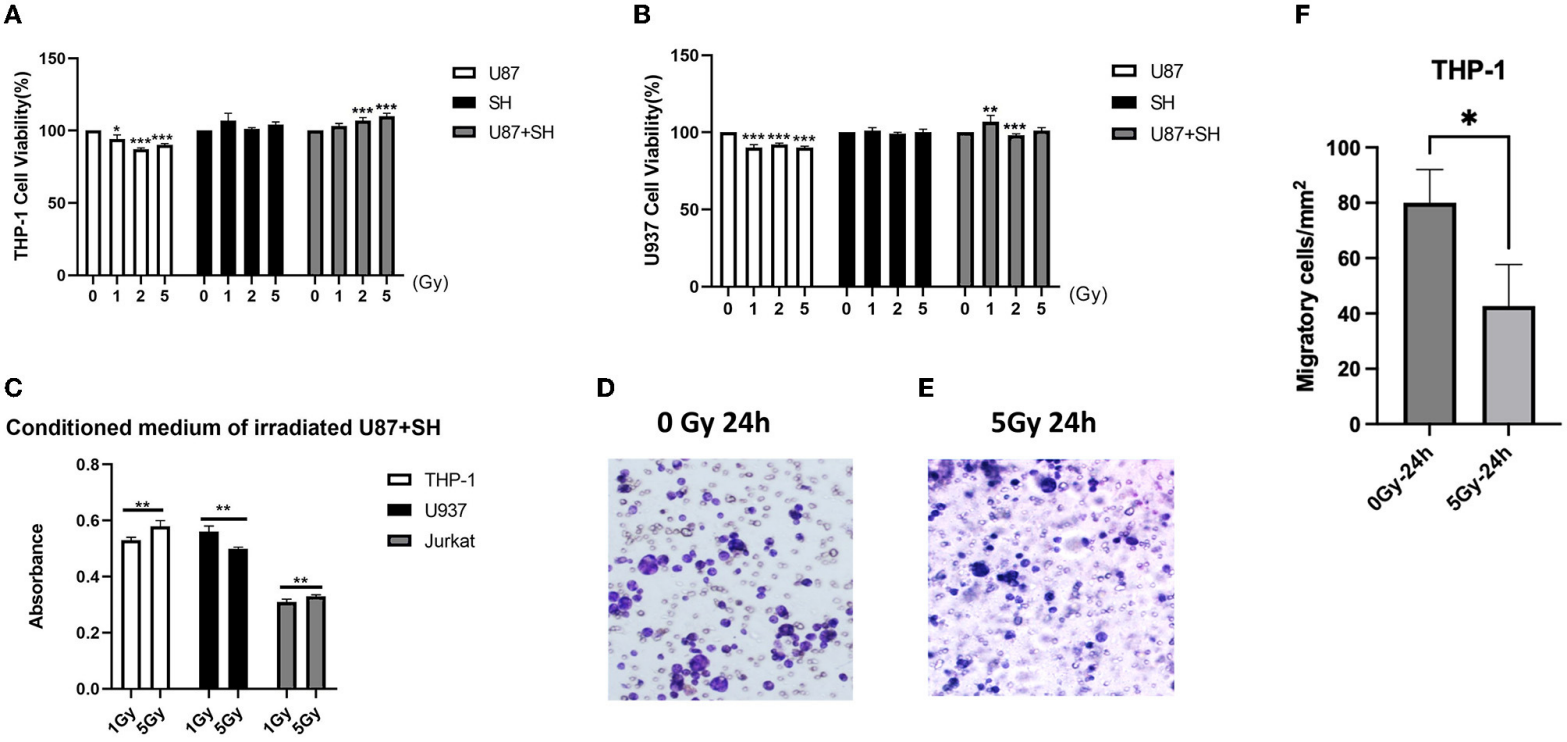
Heavy ion-irradiated neural cells mediate immune effects in vitro. **(A)** THP-1 cells (*n* = 3/g), **(B)** U937 cells (*n* = 3/g) were cultured in three different types of conditioned medium, and cell proliferation was determined after 24 h. **(C)** The MTT assay of the conditioned medium after heavy ion irradiation showing different dose-dependent effects on different immune cells. **(D, E)** Representative illustration of THP-1 in transwells (bar = 100 μM) (*n* = 3/g). **(F)** Quantification of THP-1 cell migration (*n* = 3/g). Data are shown as mean + SD in control (three biological replicates), radiated groups (three biological replicates), and three technical replicates using an unpaired two-tailed Student's *t*-test and one-way ANOVA test. The differences were considered statistically significant at the value of p. ^*^p < 0.05, ^**^p < 0.005 vs. control. U87, glial cells: SH, neuronal cell: U87 + SH, glial +neuronal cells:/g, per group.

The authors apologize for these errors and state that this does not change the scientific conclusions of the article in any way. The original article has been updated.
