# Supplementary material for: Altered expression of inflammation-associated molecules in striatum: an implication for sensitivity to heavy ion radiations
Source: Front Cell Neurosci. 2023 Dec 1;17:1252958. doi: 10.3389/fncel.2023.1252958 (PMC10725200; doi:10.3389/fncel.2023.1252958)
Supplement: Supplementary file 1 [file Data_Sheet_1.docx]

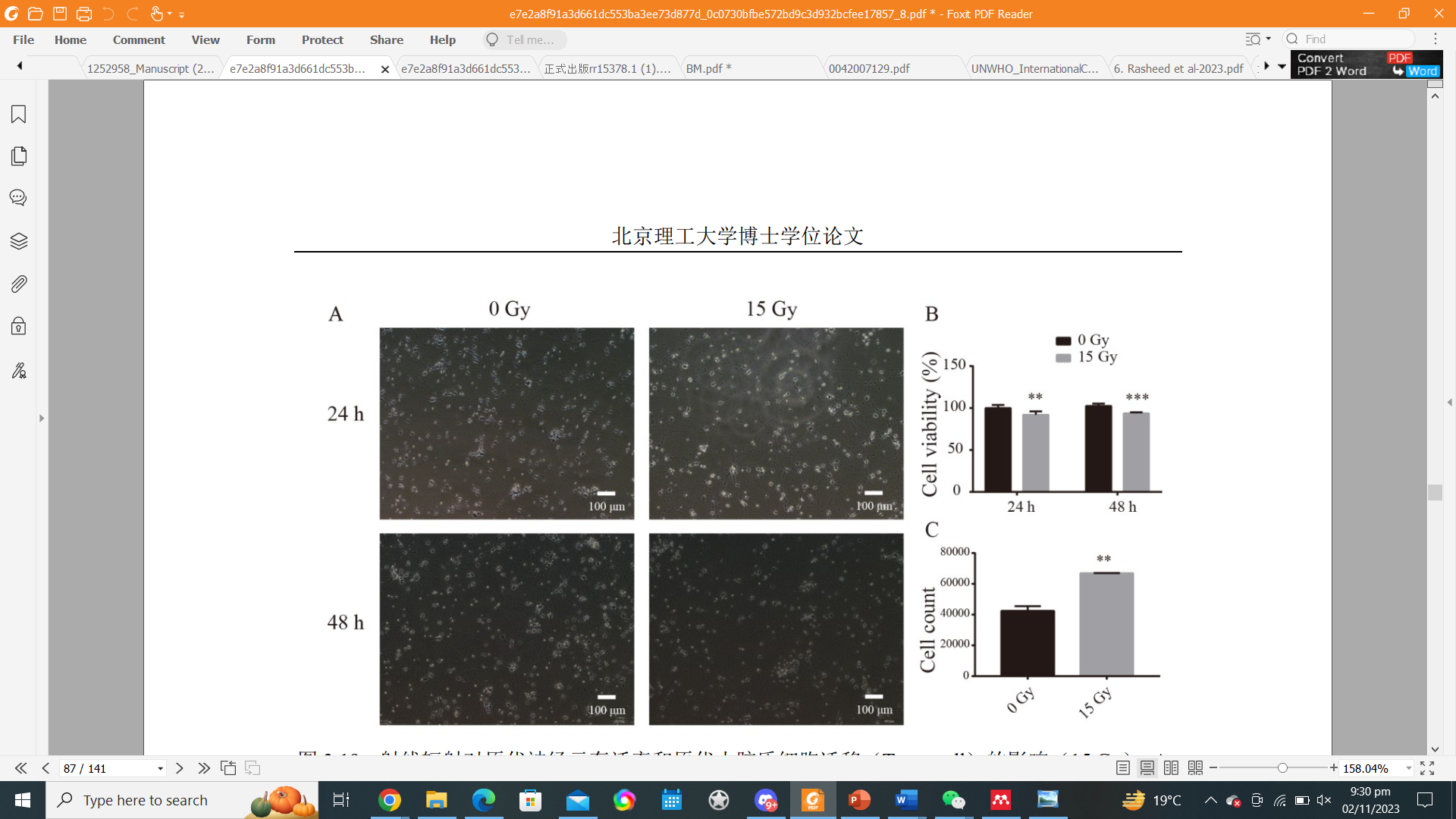


**Supplementary Figure 1:** Effect of the survival rate of primary neurons and the migration of primary microglial cells (Transwell) by γ rays (15 Gy). A: Rat primary neurons for 24 hours and 48 hours of irradiation; B: Survival rate of rat primary neurons for 24 and 48 hours of irradiation; C: Primary microglial cells after damage to primary neurons after irradiation Plasma cell migration. Ruler = 100 μm. Compared with the corresponding control group, ** represents p <0.01, and *** represents p <0.001.
